# Prdm13 forms a feedback loop with Ptf1a and is required for glycinergic amacrine cell genesis in the *Xenopus* Retina

**DOI:** 10.1186/s13064-017-0093-2

**Published:** 2017-09-01

**Authors:** Nathalie Bessodes, Karine Parain, Odile Bronchain, Eric J. Bellefroid, Muriel Perron

**Affiliations:** 1 ULB Neuroscience Institute (UNI), Université Libre de Bruxelles (ULB), B-6041 Gosselies, Belgium; 2 Paris-Saclay Institute of Neuroscience, CNRS, Univ Paris Sud, Université Paris-Saclay, UMR 9197- Neuro-PSI, Bat. 445, 91405 ORSAY Cedex, France; 3 Centre d’Etude et de Recherche Thérapeutique en Ophtalmologie, Retina France, Orsay, France

**Keywords:** Retina, Amacrine cells, Subtype specification, Prdm13, Ptf1a

## Abstract

**Background:**

Amacrine interneurons that modulate synaptic plasticity between bipolar and ganglion cells constitute the most diverse cell type in the retina. Most are inhibitory neurons using either GABA or glycine as neurotransmitters. Although several transcription factors involved in amacrine cell fate determination have been identified, mechanisms underlying amacrine cell subtype specification remain to be further understood. The Prdm13 histone methyltransferase encoding gene is a target of the transcription factor Ptf1a, an essential regulator of inhibitory neuron cell fate in the retina. Here, we have deepened our knowledge on its interaction with Ptf1a and investigated its role in amacrine cell subtype determination in the developing *Xenopus* retina.

**Methods:**

We performed *prdm13* gain and loss of function in *Xenopus* and assessed the impact on retinal cell fate determination using RT-qPCR, in situ hybridization and immunohistochemistry.

**Results:**

We found that *prdm13* in the amphibian *Xenopus* is expressed in few retinal progenitors and in about 40% of mature amacrine cells, predominantly in glycinergic ones. Clonal analysis in the retina reveals that *prdm13* overexpression favours amacrine cell fate determination, with a bias towards glycinergic cells. Conversely, knockdown of *prdm13* specifically inhibits glycinergic amacrine cell genesis. We also showed that, as in the neural tube, *prdm13* is subjected to a negative autoregulation in the retina. Our data suggest that this is likely due to its ability to repress the expression of its inducer, *ptf1a*.

**Conclusions:**

Our results demonstrate that Prdm13, downstream of Ptf1a, acts as an important regulator of glycinergic amacrine subtype specification in the *Xenopus* retina. We also reveal that Prdm13 regulates *ptf1a* expression through a negative feedback loop.

## Background

The vertebrate retina is a suitable model system for studying neurogenesis. It comprises six classes of retinal cells organized in three cellular layers: retinal ganglion cells and displaced amacrine cells in the ganglion cell layer (GCL); bipolar, amacrine and horizontal cells as well as Müller glia in the inner nuclear layer (INL); rod and cone photoreceptors in the outer nuclear layer (ONL). These cell classes can be further divided into over more than 60 different subtypes of neurons. Amacrine cells belong to the most diverse class, with about 30 morphologically distinct subtypes [1, 2], and show a high molecular diversity [3]. Numerous cell fate determinants have been identified in the different classes of retinal cells [4–7]. Yet, the mechanisms by which retinal cell subtypes diversity is generated during development remain poorly understood.

Despite their broad morphological diversity, amacrine neurons are often divided in only two groups based on the expression of inhibitory glycine or γ-aminobutyric acid (GABA) neurotransmitters. These two subtypes have distinct birthdates, GABAergic amacrine cells being generated earlier than glycinergic ones [3, 8]. This suggests that different genetic programs are used to determine these cellular subtype identities. It is thus important to examine cell-fate determination at the level of retinal cell subtypes. Some studies have addressed this issue and identified factors involved in amacrine subtype specification such as Neurod6, Bhlhb5 (Bhlhe22), Barhl2, Nr4a2, Islet-1 (Isl1), Ebf, [9–16]. We here focused our interest on the PRDM (PRDI-BF1 and RIZ homology domain) family of transcription factors.

PRDM proteins are characterised by a variable number of zinc-finger domains and a PR (PRDI-BF1-RIZ1) domain related to the SET (Su(var)3–9, Enhancer-of-zeste and Trithorax) domain found in a large group of histone methyltransferases [17, 18]. PRDM family members emerged as important regulators of neural development. In the retina, Prdm1 (Blimp1) was shown to specify photoreceptor over bipolar neuronal fate [19, 20]. Similarly to Isl1 and Bhlhb5, Prdm8 is part of the regulatory network governing bipolar cell development and amacrine cell diversity [21]. Interestingly, mutations in *prdm8* can cause human congenital stationary night blindness [21]. In the dorsal spinal cord, Prdm13 regulates neuronal diversity as a direct downstream target of Ptf1a (Pancreas Specific Transcription Factor, 1a) [22, 23]. Ptf1a is a bHLH (basic helix loop helix) transcription factor that determines inhibitory over excitatory neuronal identity in the spinal cord [24, 25], the cerebellum [26, 27] and the retina [28–33]. In the mouse retina, Prdm13 regulates subtype specification of amacrine cells, preferentially promoting GABAergic and glycinergic identities [34]. Mutations in human *prdm13* were recently found as causative of North Carolina macular dystrophy (NCMD) [35, 36]. NCMD is an autosomal dominant disease characterized by central macular defects that are present at birth, which shares phenotypic similarity with age-related macular degeneration [37]. This disorder was initially described in a family in North Carolina, but affected individuals have also been identified in Europe, Asia and South America.

In order to gain more insights into the role of Prdm13 in amacrine cells, we investigated the impact of *prdm13* gain and loss of function in the *Xenopus* retina. First, we found that *prdm13* is expressed in a subset of retinal progenitors and remains expressed in about 40% of amacrine cells, of GABA and glycinergic identity. We found that *prdm13* knockdown leads to a dramatic decrease in glycinergic amacrine cell genesis, while GABAergic cells remain largely unaffected. *Prdm13* overexpression promotes all amacrine cells, with a bias towards a glycinergic phenotype. We also provided evidence that in the retina, *Prdm13* also functions downstream of Ptf1a, and that it is subjected to negative autoregulation, likely due to its ability to repress *Ptf1a* expression. Together, this work highlights Prdm13 as a key determinant of glycinergic amacrine cell fate.

## Methods

### *Xenopus laevis prdm13* expression construct

A *Xenopus laevis* cDNA clone containing the full *prdm13* open reading was amplified by RT-PCR using total RNA isolated from stage 40 tadpole eyes, using the following primers: forward 5′- GGAATTCCATGCATTGCAACAGGGCTC-3′ and reverse 5′-CCGCTCGAGTTAGGGTTCCTTGCTGCTTCCAG-3′. This led to the amplification of two distinct sequences (*prdm13–1* and *prdm13–2* GenBank BankIt submission ID: KY555727 and KY555728, respectively). These sequences were cloned into the EcoRI and XhoI restriction sites of the pCS2-Flag vector. In the present study, we worked with pCS2-Flag-*prdm13–2*, thereafter named pCS2-*prdm13*, since it encodes a protein showing the highest identity to the Prdm13 sequences characterised in other vertebrates.

### Embryo culture, micro-injections and animal cap assays

*Xenopus laevis* embryos were obtained from adult frogs by hormone induced egg-laying and in vitro fertilization using standard methods and staged according to Nieuwkoop and Faber (1967). Synthetic mRNAs were made using Sp6 mMESSAGE mMACHINE (Ambion) and injected in a volume of 5 nl at a concentration of 25–50 pg/nl. Templates include pCS2-*prdm13* and previously described ones: pCS2-*ptf1a-GR* [38], pCS2-Flag-*mprdm13* (mouse *prdm13*, [23]), pCS2-*GFP* and pCS2-*lacZ* [39]. Standard control- and antisense-morpholino oligonucleotides (MO) were obtained from Genetools. We used *ptf1a*, *prdm13* and *prdm13*–5-mismatched (*5 mm*-MO) antisense morpholinos as previously described [23, 38]. Of note, the specificity of both *ptf1a* and *prdm13* MOs had already been demonstrated [23, 38]. All MO were injected in a volume of 5 nl and at a concentration of 50-100 μM. Embryos were injected at the two-cell stage in both blastomeres and either fixed or frozen at −80 °C at the indicated developmental stages. Embryos were co-injected with *GFP* mRNA as a tracer for the injection. Protein activity of Ptf1a-GR was induced by addition of 10 μM dexamethasone (Sigma) to the culture medium at the indicated stages.

For animal cap assays, 50-150 pg of in vitro synthesized mRNA (*ptf1a*-GR, *lacZ* or *mprdm13*) and 20 ng of MO (*prdm13*-MO, *5 mm*-MO or control-MO) were microinjected into the animal region of each blastomere at the four-cell stage. Animal caps were dissected at the blastula stage (stage 9) and cultured in 1X Steinberg medium, 0.1% BSA until stage 26. Dexamethasone (10 μM) was added at stage 12 for Ptf1a-GR activation.

### In vivo lipofection

pCS2-*GFP* and pCS2-*prdm13* were mixed with DOTAP liposomal reaction (Roche) in a 1:3 ratio and injected at stage 18 into the presumptive region of the retina as previously described [40, 41]. Embryos were fixed at stage 41 and cryostat sectioned (12 μm). GFP-positive cells were counted and cell types were identified based on their laminar position and morphology.

### In situ hybridization and immunohistochemistry

Digoxigenin-labeled antisense RNA probes for *gad1* (also called *gad1.1*, [42]), *vglut1* (also called *slc17a7*, [43]), *glyt1* (also called *slc6a9*, [44]) and *prdm13* [23] were generated according to the manufacturer’s instruction (Roche). Whole-mount in situ hybridization analysis of *Xenopus* embryos was performed as described [45]. For sections, embryos were agarose-embedded and vibratome-sectioned at 50 μm thickness. In situ hybridization at stage 42, double fluorescent in situ hybridizations or combination of in situ hybridization and immunofluorescent staining were performed on 12 μm cryostat sections following previously described procedures [46]. For EdU experiments, stage 28/30 or 42 tadpoles were incubated 3 h in a 1 mM EdU solution, then immediately fixed in 4% paraformaldehyde. In situ hybridization was first performed followed by Edu staining using the Click-iT EdU Alexa Fluor 488 Imaging Kit (Molecular Probes).

For immunofluorescent labelling, embryos were fixed in 4% paraformaldehyde/0.3% glutaraldehyde in 0.1 M phosphate buffer, pH 7.4 for 20 min. Then, they were cryoprotected with 30% (*w*/*v*) sucrose in PBS before cryosectioning (12 μm thickness). Immunolabeling was performed using rabbit anti-glycine (1:100, AB139, Millipore or 1:500, IG1001, Immunosolution), mouse anti-GFP (1:500, A11120, Molecular probes), rabbit anti-GABA (1:1000, 20,094, Immunostar), rabbit anti-calretinin (1:100, 7697, Swant) as primary antibodies, and anti-rabbit Alexa 594 (1:1000, A11012, Molecular Probes) or anti-mouse Alexa 488 (1:1000, A11001, Molecular Probes) as secondary antibodies. To improve the signal of the glycine antibody, an antigen retrieval method was performed as previously described [47]. Images were acquired on M2 Zeiss microscope with a digital camera AxioCam MRc and AxioVision Rel 7.8 software.

### RT-qPCR analysis

Total RNA was extracted using the RNAspin Mini RNA isolation kit (GE Healthcare), cDNA was synthesized with the iScript™ _C_DNA synthesis kit (Biorad). Real time RT-qPCR reactions were performed in technical triplicates using the Step One Plus real Time PCR system (Applied biosystems) with Go Taq® qPCR Master Mix (promega) for SYBR Assay. *Xenopus gapdh* and *odc1* were used as reference genes. The following primers were used: *prdm13* (forward: 5′-CTGCCGACACATGATGAAAAAGG-3′ and reverse: 5′-AGATTTTGGGGGAGGCAGAAAAG-3′); *ptf1a* (forward: 5′-CGGACTCCTTTGGTTCCAC-3’ and reverse: 5′- CATTGGAATGATAAAGAGCGGG); *neurog2* (forward: 5′-GGCGCGTTAAAGCTAACAAC-3′ and reverse: 5′-TTCGCTAAGAGCCCAGATGT-3′); *gapdh* (forward 5′-TAGTTGGCGTGAACCATGAG-3′ and reverse 5′-GCCAAAGTTGTCGTTGATGA-3′); and *odc1* (forward: 5′- TTCTACTCGAGCAGCATTTGG-3′ and reverse: 5′-TTCAAACAACATCCAGTCTCC-3′). For animal caps experiments, 40–50 embryos were used for each point. For retina experiments, 50–60 eyes were dissected for each point.

## Results

### *Prdm13* is expressed in few progenitors and in a subset of amacrine cells during *Xenopus* retinogenesis

**Fig. 1:**
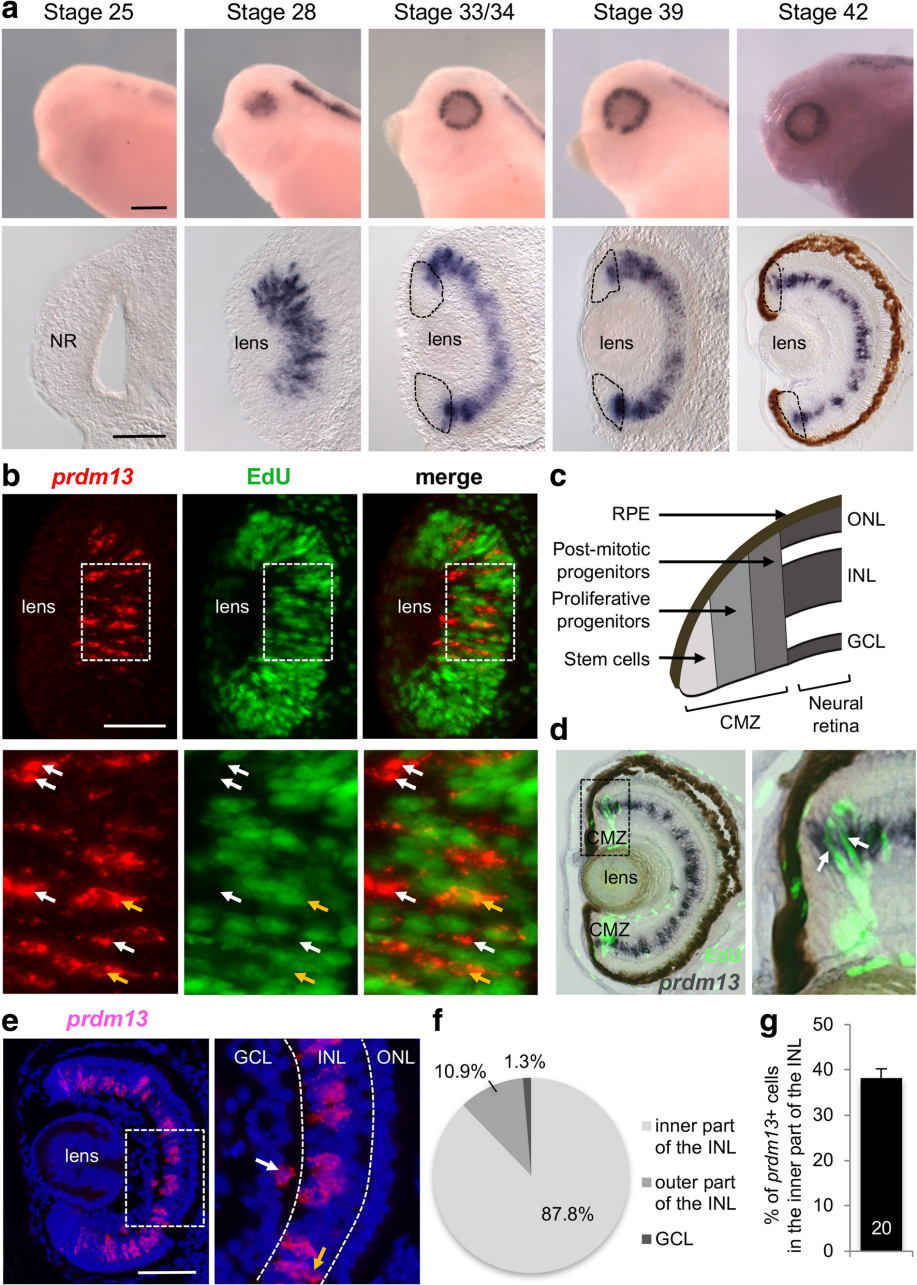
*Prdm13* expression in the *Xenopus* retina. **a** Whole-mount in situ analysis of *prdm13* expression during embryogenesis, shown as lateral views of the embryo heads or as transversal retinal sections at the indicated stages. At stage 42, the in situ hybridization has been performed on retinal section. The brown colour is the retinal pigment epithelium. Dotted lines delineate the ciliary marginal zone (CMZ). **b** Stage 28/30 sections following *prdm13* in situ hybridization (red) and EdU incorporation assay (green). Below are enlargements of areas delineated with dotted lines. White arrows point to *prdm13* ^*+*^ */*Edu^−^ cells while yellow arrows point to double labelled cells. **c** Schematic of a CMZ in the periphery of a *Xenopus* tadpole retina. **d** Stage 42 retinal section following *prdm13* in situ hybridization (dark blue) and EdU incorporation assay (green). Since EdU labels cells that are in the S-phase during the 3-h EdU pulse, not all proliferative cells are labelled, in particular the slowly cycling stem cells. Panel on the right is an enlargement of the area delineated with dotted lines. Arrows point to double labelled cells. **e** *Prdm13* fluorescent in situ hybridization (red) on stage 41 retinal section, counterstained with Hoechst to visualize nuclei (blue). Panel on the right is an enlargement of the white square. The white and yellow arrows point to *prdm13* positive cells localized in the ganglion cell layer (GCL), and the outer part of the inner nuclear layer (INL), respectively. Dotted lines delineate the three nuclear layers. **f** Pie chart showing the distribution of *prdm13* ^+^ cells among the ganglion cell layer (GCL, 1.3 ± 0.5%), the inner part of INL (87.8 ± 1.3%) and the outer part of the INL (10.9 ± 1.3%). Data are presented as mean ± SEM, *n* = 20 sections. **g** Quantification of the percentage of *prdm13* positive cells among amacrine cells (defined by their localization in the inner part of the INL). Data are presented as mean ± SEM. Number of analysed sections is indicated in the bar. NR: Neural Retina, CMZ: ciliary marginal zone; RPE: Retinal Pigmented Epithelium, GCL: Ganglion Cell Layer, INL: Inner Nuclear Layer, ONL: Outer Nuclear layer. Scale bar represents 200 μm (whole mount), 100 μm (sections)

The spatial and temporal distribution of *prdm13* transcripts during *Xenopus laevis* retinogenesis was analysed by whole-mount in situ hybridization (Fig. 1a). *Prdm13* expression is detected in the presumptive eye region from stage 28 onwards. We confirmed on transversal sections that it is not detected in the optic vesicle at stage 25. At stage 28, the optic vesicle contains both proliferative progenitor cells and early born post-mitotic precursors. In order to assess in which cell population *prdm13* was expressed, we combined EdU labelling (to visualize cells in the S-phase of the cell cycle) with *prdm13* in situ hybridization (Fig. 1b). *prdm13* labelling was mainly detected in EdU-negative cells, suggesting that it is primarily expressed in post-mitotic retinal cells. Yet, we found 25.17% (±1.97, *n* = 10 sections, 305 cells) of double-labelled EdU/*prdm13* cells among the *prdm13* ^+^ cell population. From stage 33/34, *prdm13* expression is restricted to the INL of the retina and to the ciliary marginal zone (CMZ), where continuous neurogenesis occurs [48]. Within the CMZ, cells are spatially ordered, so that retinal stem cells reside in the most peripheral region, proliferating progenitor more centrally and post-mitotic precursors in the most central region [49] (Fig. 1c). *prdm13* expression was not detected in the tip of the CMZ, suggesting that it is not expressed in retinal stem cells. In order to discriminate its expression in proliferative versus non-proliferative cells, we combined *prdm13* in situ hybridization and EdU labelling. We found double-labelled cells in the central region of the CMZ suggesting that at least a subset of proliferative progenitors express *prdm13* (Fig. 1d). At post-embryonic stage 42, in addition to this expression in the CMZ, the expression in the INL gets mostly restricted to the inner part of the layer. To further identify and quantify *prdm13* cell populations, we performed fluorescent in situ hybridization on retinal sections that allows assessing transcripts expression at a cellular resolution (Fig. 1e). We first quantified the distribution of *prdm13* ^+^ cells among the different layers of the retina (Fig. 1f). Most of *prdm13* ^+^ cells (87.8%) are localized in the inner part of the INL (two cell body rows), where most of the amacrine cells reside. Some (10.9%) are also found in the outer part (one cell body row), where cell bodies of bipolar, horizontal and Müller cells are located. Of note, scattered amacrine cells can occasionally be found in the outer part of the INL at this stage (as inferred from staining using amacrine cell markers, data not shown). Finally, a small percentage (1.3%) are located in the GCL. We then quantified the number of *prdm13* ^+^ cells among the amacrine cell population (Fig. 1g). We found that 38,3% of amacrine cells are *prdm13* positive. Together, these data indicate that *prdm13* is expressed both in a small subset of retinal progenitors (in the optic cup and in the CMZ) and in about a third of amacrine cells.

### *Prdm13* is expressed in glycinergic and GABAergic amacrine cells

**Fig. 2:**
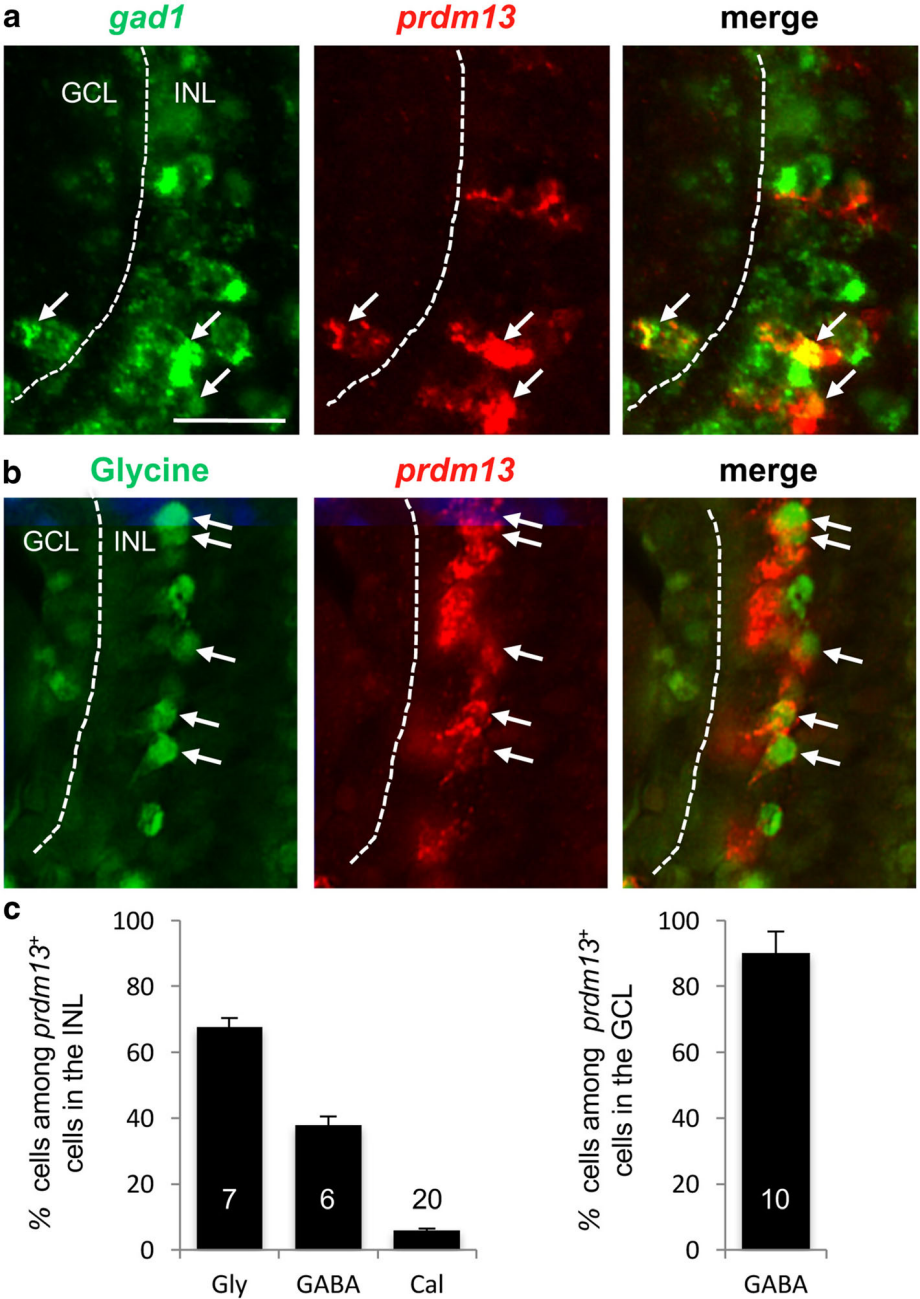
*Prdm13* is expressed in glycinergic- and GABAergic-amacrine cells. **a** Double in situ hybridization with *prdm13* (red) and *gad1* (green) probes on stage 39/40 retinal section. **b**In situ hybridization with *prdm13* probe (red) coupled with anti-Glycine immunostaining (green) on stage 41/42 retinal section. Arrows point to double labelled cells. Dotted lines separate the GCL and the INL. **c** Quantification of the percentages of GABA (*gad1* ^+^), Glycine (Gly) and Calretininin (Cal) amacrine cells among *prdm13* ^+^ cells in the INL and in the GCL. Data are presented as mean ± SEM. Number of analysed sections is indicated in each bar. GCL: Ganglion Cell Layer, INL: Inner Nuclear Layer. Scale bar represents 25 μm

Amacrine cells are inhibitory neurons that mainly use GABA or glycine as neurotransmitters. To investigate in which amacrine cell subtypes *prdm13* is expressed, we first performed double fluorescent in situ hybridizations with *prdm13* and *gad1* (glutamate decarboxylase 1) as a marker for GABAergic neurons [42]. We found that 38% of *prdm13* cells within the INL were *gad1* positive (Fig. 2a,c). Interestingly, we found that among the *prdm13* expressing cells located in the GCL, 90% are *gad1* ^+^, strongly suggesting that these cells are displaced amacrine cells. However, although unlikely, we cannot completely rule out that these are GABAergic ganglion cells as expression of GABA in retinal ganglion cells has been observed in some species [50, 51]. Besides, whether all displaced amacrine cells are GABAergic in *Xenopus* has not yet been determined. *prdm13*-positive cells that are localized in the GCL and are GABA-negative may thus be non-GABAergic displaced amacrine cells. We then combined *prdm13* in situ hybridization with anti-glycine or anti-calretinin immunostaining (Fig. 2b and data not shown). We found that 68% of *prdm13* cells were glycine^+^ while only 5% were calretinin^+^ (Fig. 2c). Of note, it has been shown in *Xenopus* retina that 80–90% of calretinin positive amacrine cells are also GABAergic [52]. Together, these results show that *prdm13* is primarily expressed in glycinergic and GABAergic subtypes of amacrine cells.

### *Prdm13* overexpression promotes amacrine cell fate with a bias towards a glycinergic phenotype

**Fig. 3:**
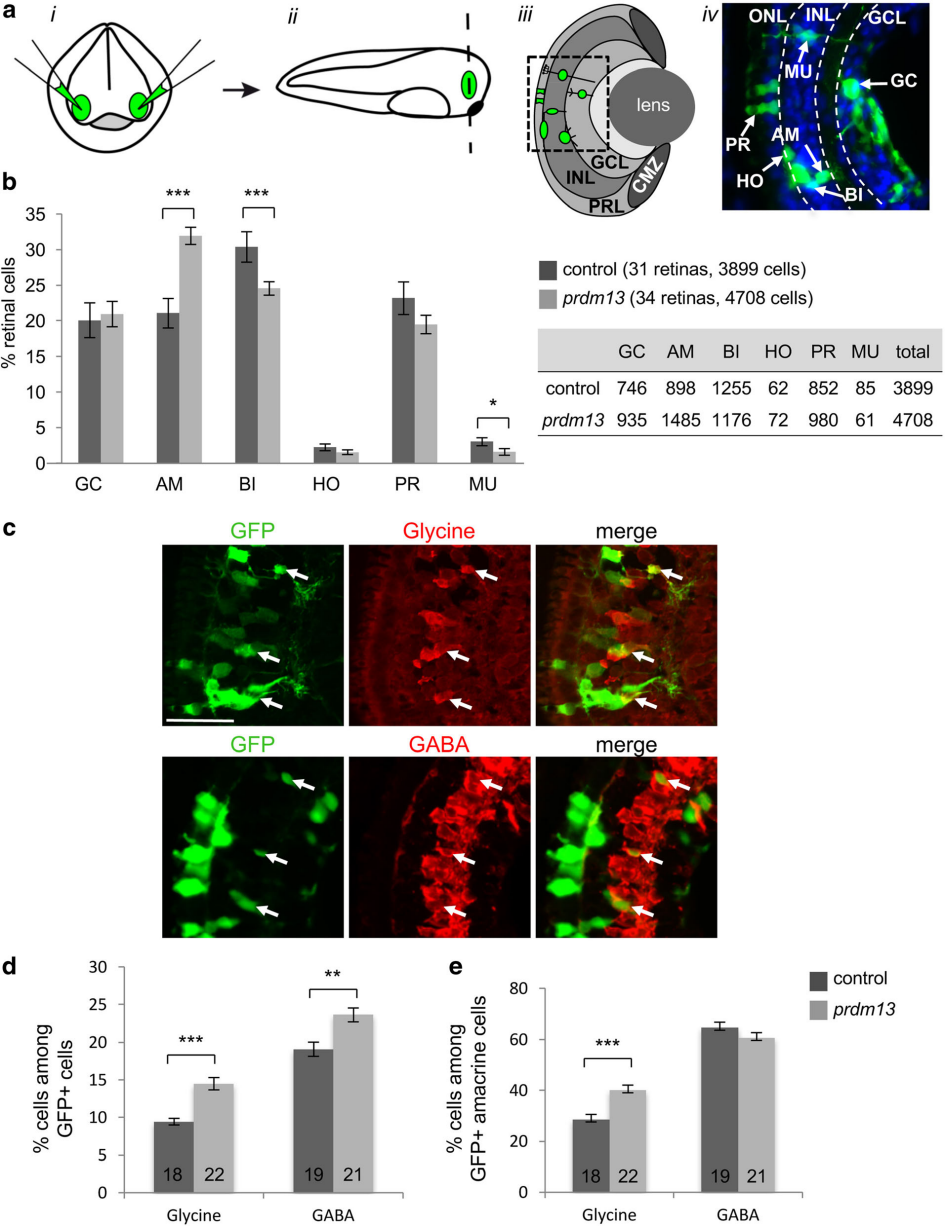
*Prdm13* overexpression promotes amacrine cells with a bias toward a glycinergic cell fate. **a** Illustration of the lipofection technique. ***i*** DNA is injected in the eye fields (green) of stage 18 embryos (frontal view). ***ii*** Retinas (green) are then sectioned (dashed line) at stage 41. ***iii*** Schema of a retinal section showing a clone of transfected cells (green) in the different retinal layers. ***iv*** Picture of a retinal section area (square in c) showing transfected cells (green). Nuclei are counterstained with Hoechst (blue). **b** Proportion of different retinal cell types in stage 41 *prdm13* lipofected and control embryos. The table indicates the absolute numbers of counted cells for each cell type. **c** Double-immunostaining with anti-GFP and anti-Glycine or anti-GABA antibodies on retinal sections of *prdm13* lipofected embryos. Arrows point to GFP positive cells that are Glycine or GABA-positive. **d,e** Quantification of GABA-positive and Glycine-positive cells among total GFP^+^ cells (**d**) or among GFP^+^ cells in the inner part of the INL where amacrine cells reside (**e**). Number of analysed retinas is indicated in each bar. GC: ganglion cells; AM: amacrine cells; BI: bipolar cells; HO: horizontal cells; PR: photoreceptor cells; MU: Müller cells. Values are given as mean ± SEM. * *p*-value <0,05; ** *p*-value <0,01; *** *p*-value <0,001 (Mann-Whitney test). Scale bar represents 50 μm

To investigate the involvement of Prdm13 in neuronal specification within the retina, we first used a gain of function approach. As no cDNA containing the entire *Xenopus laevis prdm13* open reading frame was available, we cloned the full-length *prdm13* cDNA. We then overexpressed *prdm13* in 2-cell stage embryos through mRNA injections. As previously described, when the mouse *prdm13* mRNA was overexpressed in *Xenopus* embryos (*mprdm13*, [23]), we found that overexpressing *Xenopus prdm13* mRNA leads to gastrulation defects preventing subsequent analysis of retinal development (data not shown). We thus decided to overexpress *prdm13* only in a subset of retinal progenitors by in vivo lipofection in the eye field at stage 18. GFP expressing plasmid was co-transfected and used as a tracer to identify transfected cells at stage 41, when cells in the central retina are differentiated (Fig. 3a). *Prdm13* clones exhibited an increased proportion of amacrine cells at the expense of bipolar and Müller cells, compared to control retina from embryos lipofected with GFP alone (Fig. 3b). To know whether a particular amacrine cell subtype was favoured, we combined in vivo lipofection with anti-GABA or anti-glycine immunostaining (Fig. 3c). We found that the proportion of both GABAergic and glycinergic amacrine cells among lipofected cells is increased (Fig. 3d). However, the proportion of GABAergic neurons among the transfected amacrine cell population is not significantly affected, while the proportion of glycinergic neurons is higher than in controls (Fig. 3e). Together, these data suggest that Prdm13 acts cell autonomously in retinal precursors to promote GABAergic and glycinergic amacrine cell genesis, with a bias towards a glycinergic phenotype.

### *Prdm13* loss of function leads to a decrease in glycinergic amacrine cell genesis

**Fig. 4:**
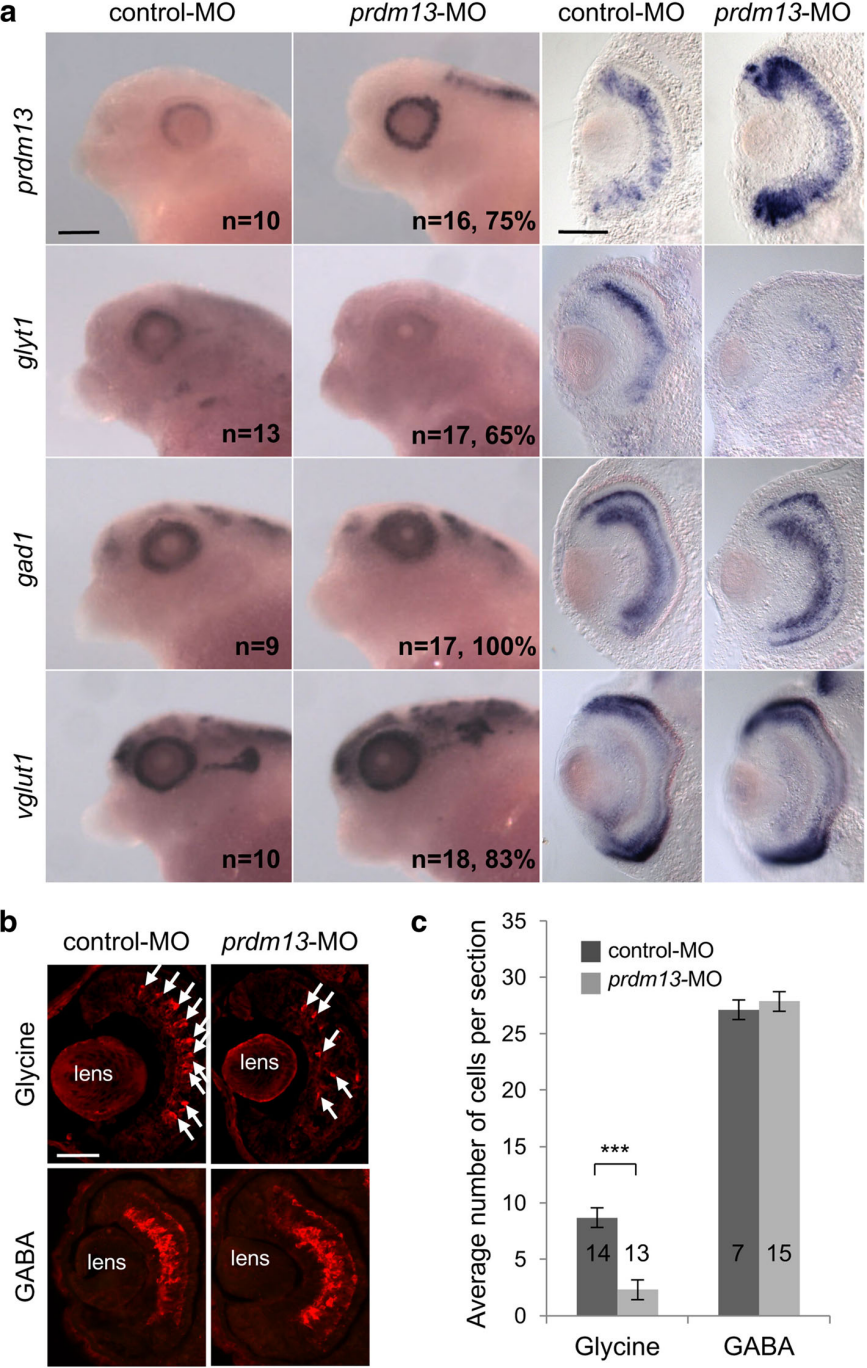
*Prdm13* loss of function leads to a decrease in glycinergic but not GABAergic-amacrine cells. **a** Whole-mount in situ hybridization analysis of *prdm13*, *glyt1*, *gad1* and *vglut1* expression on stage 39/40 embryos injected with *prdm13*-M0 or control-MO. Lateral views of the head and transversal sections of the retinas are shown. The number of analysed embryos and the percentage of embryos with represented phenotypes are indicated in each panel. **b** Stage 39/40 sections following GABA or Glycine-immunostaining on control-MO and *prdm13*-MO injected embryos. Arrows point to Glycine-positive cells. **c** Quantification of the average number of GABA- or Glycine-positive cells per section. Number of analysed sections is indicated in each bar. Data are presented as mean ± SEM. *p* < 0.001 (***) (Mann-Whitney test). Scale bars represent 200 μm (whole mount), 100 μm (sections)

To address the potential requirement of Prdm13 in amacrine cell genesis, we performed loss of function experiments using a previously designed *prdm13* translation blocking morpholino antisense oligonucleotide (*prdm13*-MO, [23]) that targets both *Xenopus laevis prdm13* alloalleles. *Prdm13*-MO or a control-MO were injected in two blastomeres at two-cell stage, and retinal phenotypes were analysed by whole mount in situ hybridization at stage 41. As the knockdown of *prdm13* was previously shown to strongly upregulate *prdm13* expression in the dorsal neural tube [23], we first tested the effect of the injection of *prdm13*-MO on the expression of *prdm13* itself. We found that *prdm13*-MO injection leads to endogenous *prdm13* mRNA upregulation (Fig. 4a), indicating that Prdm13 also negatively regulates its expression in a feedback loop in the retina. Probes for *gad1*, *vglut1* (vesicular glutamate transporter 1, [42]), and *glyt1* (glycine transporter 1, [44]) were next used as markers of GABAergic, glutamatergic and glycinergic neurons, respectively. Whereas *vglut1* and *gad1* stainings were not affected by *prdm13*-MO injection, *glyt1* expression was dramatically reduced in the retina (Fig. 4a). This effect was quantified by immunostaining experiments using anti-glycine or anti-GABA antibodies (Fig. 4b-c). A significant decrease in the number of glycinergic cells following *prdm13*-MO injection was observed compared to control-MO injected embryos, while no effect was observed regarding GABAergic amacrine cell labelling. Together, these data reveal that *prdm13* knock-down impacts glycinergic, but not GABAergic amacrine cell genesis.

### Prdm13 is a Ptf1a target in *Xenopus* retinal progenitor cells

**Fig. 5:**
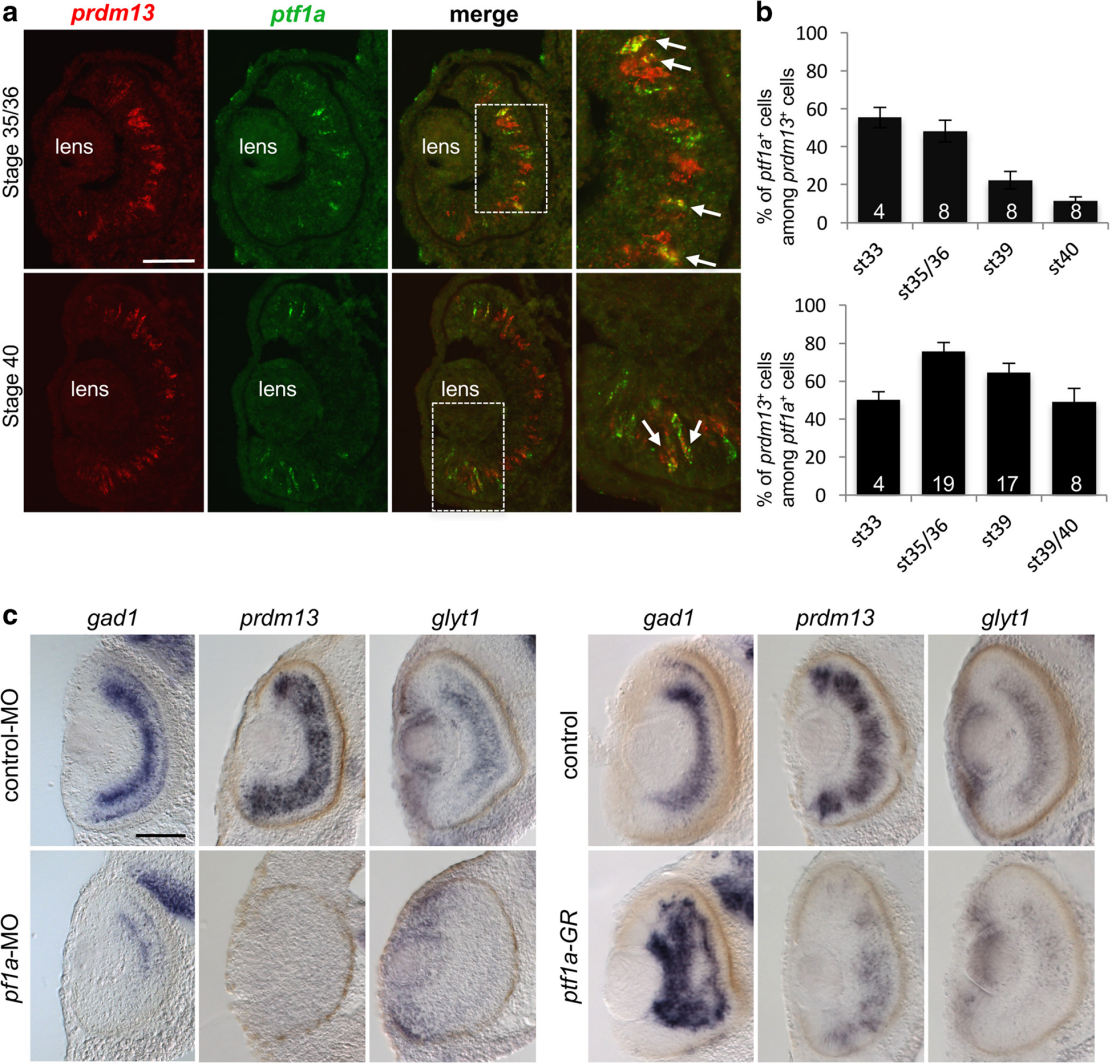
*Prdm13* expression in the retina upon *Ptf1a* gain and loss of function. **a** Double fluorescent in situ hybridization with *prdm13* (red) and *ptf1a* (green) probes on wild type retina at stage 35/36 and 40. Right panels are enlargement of central or peripheral retina (white squares). Arrows show double labelled cells. **b** Quantification of the percentage of *ptf1a* ^*+*^ cells among the *prdm13* ^*+*^ cell population (top graph) and the percentage of *prdm13* ^*+*^ cells among the *ptf1a* ^*+*^ cell population (bottom graph) at different stages of retinogenesis. Data are presented as mean ± SEM. Number of analysed sections is indicated in each bar. **c** Analysis of *gad1*, *prdm13* and *glyt1* expression on stage 41 retinal transversal sections following whole mount in situ hybridization on embryos injected with *ptf1a*-MO, control-MO, *GFP* mRNA (control) or *ptf1a-GR* mRNA. Dexamethasone was added at stage 21/22 to activate the Ptf1a-GR fusion protein. Scale bar represents 100 μm

*Prdm13* is a direct target of Ptf1a in the dorsal neural tube [22, 23]. To determine the interaction between these two factors in the retina, we first investigated whether both genes are co-expressed in retinal cells, using double fluorescent in situ hybridizations (Fig. 5a). The percentage of double-labelled cells was then calculated at different stages of retinogenesis (Fig. 5b). At stage 33, about 50% of *prdm13* ^+^ cells are *ptf1a* ^+^, and vice versa. The percentage of *prdm13* ^+^ cells among *ptf1a* ^+^ cell population remains stable over the entire retina between stage 33 and stage 40 (50–70%). However, the number of *ptf1a* ^+^ cells among *prdm13* expressing cells progressively decreases from stage 33 to stage 40 as *ptf1a* expression gets restricted to the CMZ compartment. By stage 40 in the central retina, only 10% of *prdm13* ^+^ cells are still *ptf1a* ^+^. Thus, at all stages examined, some retinal cells express only *prdm13*, some only *ptf1a,* and some express both genes, consistent with a possible genetic interaction in these cells. Moreover, the number of co-labelled cells might be higher if the analysis had been done at the protein level since Ptf1a protein may be retained in the cells after its mRNA downregulation.

Could Prdm13 be a Ptf1a target in a subset of retinal cells? *Prdm13* expression was shown to be lost in the mouse *ptf1a* ^*−/−*^ retina [34]. To further address this question in *Xenopus*, we performed *ptf1a* gain or loss of function experiments and analysed the impact on *prdm13* retinal expression at stage 41 (Fig. 5c). We used previously described MO to generate *ptf1a* knock-down embryos [30]. *Gad1* probe was used as a readout of *ptf1a*-MO activity, as *gad1* expression was previously shown to be regulated by Ptf1a [30]. In *ptf1a* morphants, *prdm13* expression was dramatically decreased in the retina. Of note, this was also the case for *glyt1* expression.

To overexpress *Ptf1a*, we injected at the two-cell stage mRNAs encoding a glucocorticoid inducible form of Ptf1a (Ptf1a-GR) [30]. Dexamethasone (dex) was added to the embryo culture medium at stage 21/22 in order to activate Ptf1a-GR at an early stage of retinogenesis. We confirmed that under such conditions *gad1* expression is strongly upregulated at stage 41, as expected from our previous work [30] (Fig. 5c). Surprisingly, *prdm13* expression was at that stage clearly decreased compared to the controls. Since our above data suggest that Prdm13 is required for glycinergic neuron genesis, we also examined *glyt1* expression and found that it is also reduced upon *ptf1a* overexpression.

**Fig. 6:**
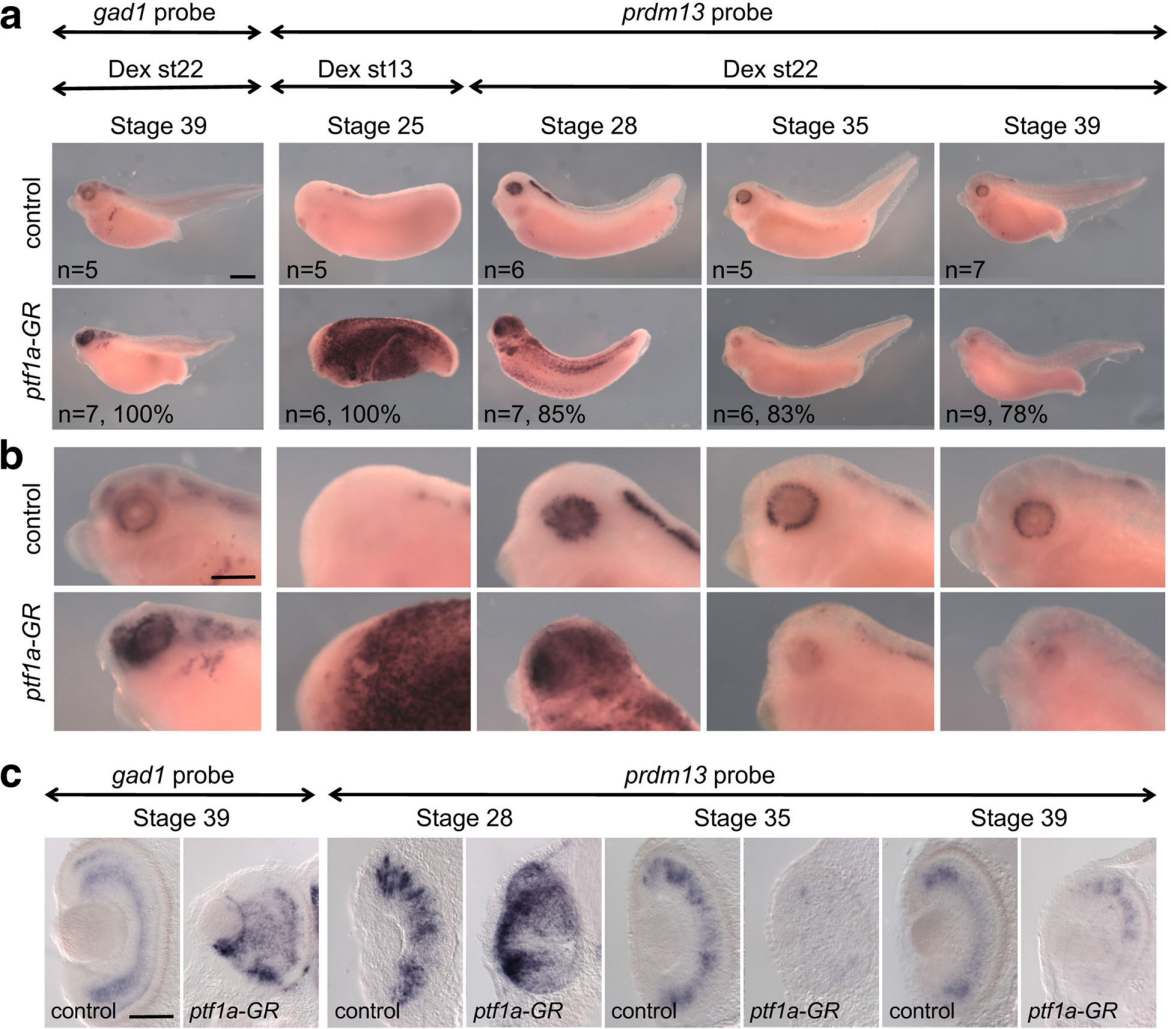
*Prdm13* expression is deregulated upon *Ptf1a* gain of function. Whole-mount in situ hybridization analysis of *prdm13* and *gad1* expression in *ptf1a-GR* mRNA injected embryos treated with dexamethasone (Dex) and analysed at the indicated stages. Shown are lateral views of the embryos (**a**), of the head at higher magnification (**b**), and transversal sections of the retinas (**c**). The number of analysed embryos and percentage of embryos with represented phenotype are indicated in each panel. Scale bars represent 400 μm (**a**, **b**) or 100 μm (**c**)

Based on these unexpected observations, we further investigated the impact of *ptf1a* overexpression on *prdm13* at different stages of development. We found robust ectopic *prdm13* expression in the epidermis of embryos at stage 25 and stage 28 (Fig. 6a,b). Importantly, in transversal sections, a strong *prdm13* upregulation was also seen at both stages in the optic vesicle and optic cup (data not shown and Fig. 6c). However, the opposite effect, i.e. decrease in *prdm13* retinal expression, was obtained from stage 35 onwards (Fig. 6a-c). Since we saw above that Prdm13 negatively regulates its own expression, this data likely reveals a feedback control mechanisms. Together, our loss and gain of function analysis suggest that *prdm13* is a target of Ptf1a in *Xenopus* retinal progenitor cells.

### Prdm13 negatively regulates *Ptf1a* in a feedback loop

**Fig. 7:**
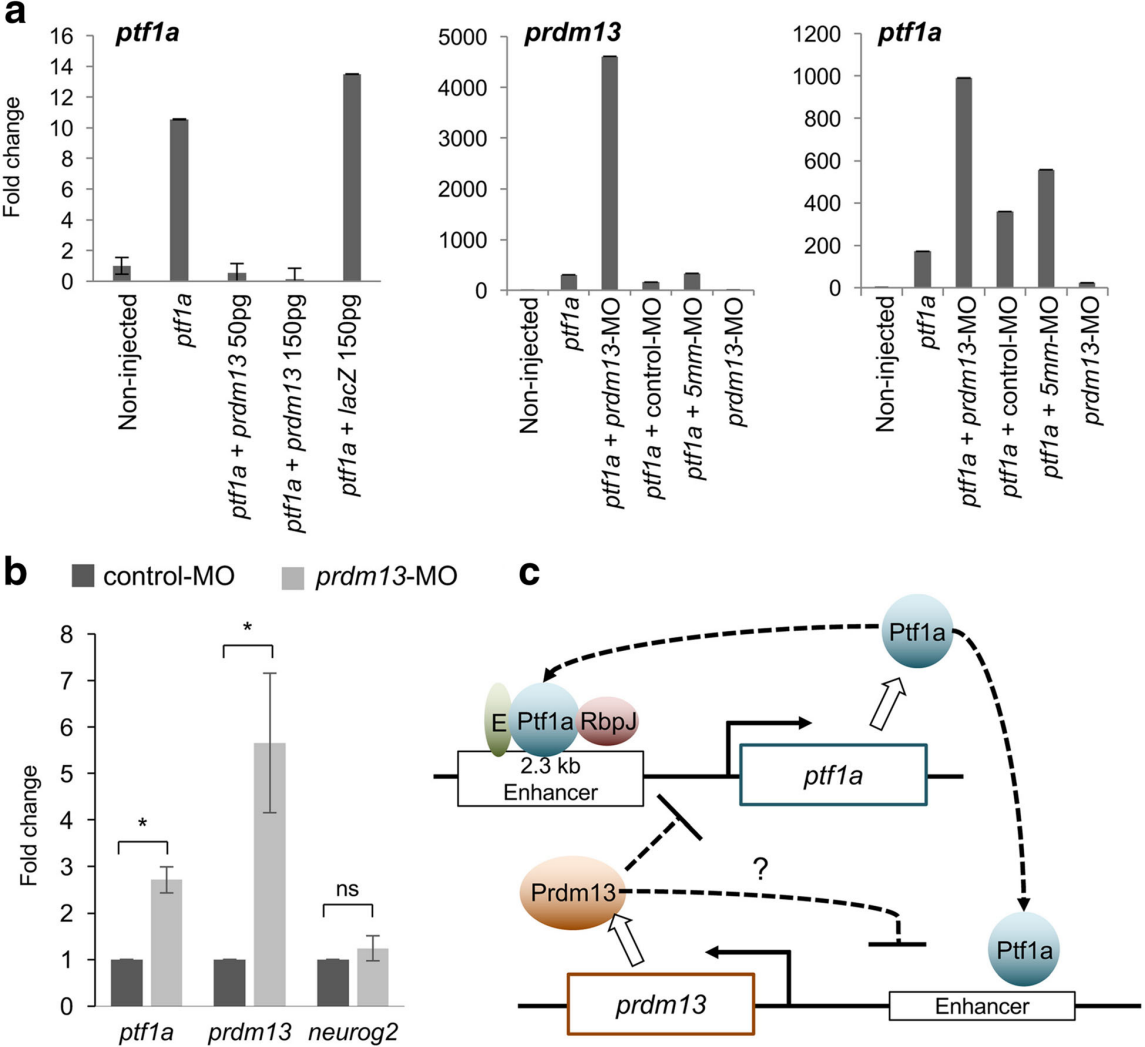
Prdm13 negatively regulates *Ptf1a* in a feedback loop. **a** RT-qPCR analysis of *prdm13* and *ptf1a* expression in animal cap explants isolated from embryos injected with *ptf1a-GR, mprdm13, lacZ* mRNA and morpholinos as indicated, and collected when sibling embryos reach stage 26. Expression levels in non-injected caps have been set to 1. Shown are representative results of one out of two independent experiments. Data are presented as means of technical triplicates ± SD. **b** RT-qPCR analysis of *ptf1a, prdm*13 *and neurog2* expression in stage 39/40 dissected eyes from control-MO or *prdm13*-MO injected embryos. Expression level in control caps has been set to 1. The graph represents a pool of 3 to 4 experiments. Data are presented as mean ± SEM. *p* < 0.05 (*) (Mann-Whitney test). **c** Drawing illustrating the interactions between *ptf1a* and *prdm13* suggested by our results. As in the neural tube [22], we found that Ptf1a positively regulates *prdm13* expression. It has previously been shown that Ptf1a binds, along with an E protein and Rbpj (PTF1-J complex), to a conserved 2.3 kb sequence located 13.4 kb 5′ to the *ptf1a* coding region and regulates its own transcription [53]. We showed here that Prdm13 negatively regulates its own expression through a negative retro-control of *ptf1a* expression. The underlying mechanism remains to be investigated. Our results also do not exclude the possibility that Prdm13 could in addition directly repress its own expression

The results above indicate that *prdm13* in the retina is subjected to negative autoregulation (Fig. 4a). This regulation could be a direct autorepressive action of Prdm13 on its own promoter. Alternatively, it could be an indirect negative control of Prdm13 on the expression of its inducer, *ptf1a*. To test this hypothesis and given that Ptf1a induces its own expression [53], we examined the effect of Prdm13 gain and loss of function on *ptf1a* gene regulation in Ptf1a-GR overexpressing animal caps. Explants were treated with Dex at stage 10, cultured until stage 26 and analysed by RT-qPCR using 3’UTR *ptf1a* primers to specifically detect endogenous *ptf1a* mRNAs. As previously reported [53], we found that Ptf1a induces its own expression (Fig. 7a). Interestingly, this upregulation of *Ptf1a* expression was abolished following *Prdm13* overexpression. Overexpression of *lacZ,* which serves as a control, had no effect on Ptf1a auto-activation. Conversely, as observed for *prdm13* expression, a stronger upregulation of *ptf1a* was observed upon *prdm13* inhibition. Such increase in the expression of *ptf1a* was not observed with a control-MO or *5 mm*-MO. These results indicate that Prdm13 regulates Ptf1a in a negative feedback loop. To determine whether this mechanism also occurs during eye development, we analysed *ptf1a* expression at stages 39/40 by RT-qPCR in dissected eyes from *prdm13*-MO or control-MO injected embryos. Both *prdm13* and *ptf1a* were upregulated in the retina of *prdm13*-MO injected embryos compared to controls (Fig. 7b). No such upregulation was observed for another bHLH gene, neurog2, that is expressed in retinal progenitors [49, 54]. Thus, Prdm13 appears to negatively retro-control *ptf1a* expression during retinogenesis.

## Discussion

Amacrine cells are the most diverse class of neurons in the retina, with over 30 different subtypes. The genetic network governing the determination of these amacrine cell subtypes remains poorly known. In the current study, we found that the transcriptional regulator Prdm13 is expressed in subtypes of amacrine cells in the *Xenopus* retina, in both glycinergic and GABAergic ones. Our gain of function analysis indicates that Prdm13 is an inducer of amacrine cells, with a bias towards a glycinergic destiny. *prdm13* knock-down prevents glycinergic cell genesis but does not significantly affect GABAergic amacrine cell specification. By combining studies in animal caps and in the retina, we also propose a regulatory feedback loop between Prdm13 and Ptf1a where the latter would promote the expression of *Prdm13*, which would then negatively retro-controls *Ptf1a* expression.

It was shown in the mice that the major population of Prdm13^+^ amacrine cells express calbindin and calretinin, two calcium-binding proteins [34]. In *Xenopus* however, calbindin is a specific marker of cone photoreceptor cells, as it is in the human retina [55]. We therefore only tested co-expression between *prdm13* and calretinin. *In Xenopus*, calretinin expression is found mainly in bipolar cells, ganglion cells and only in few amacrine cells [52], while it is found in amacrine and ganglion cells in the mouse [56]. Our data revealed only few (5%) calretinin^+^ cells among *prdm13* ^+^ population compared to 65% in the mouse [34]. Given that this calcium-binding protein labels different populations of retinal cells in different species, this apparent difference may not reveal significant difference regarding Prdm13 expression in amacrine cell subpopulations. More meaningfully, it was reported in the mouse retina that almost all of the Prdm13 amacrine cells are GABAergic (13.5%) or glycinergic (87.1%) [34]. We also found in the *Xenopus* retina that *prdm13* cells are primarily GABAergic (38%) and glycinergic (68%). In the mouse retina, it was reported that only rare *prdm13* ^*+*^ cells were proliferative cells, suggesting that *prdm13* is primarily expressed in postmitotic cells [34]. We investigated this issue at the level of *prdm13* mRNA. We also found that *prdm13* is mainly expressed in postmitotic cells. Yet, some *prdm13* ^*+*^ cells in the optic vesicle and in the CMZ were found to be EdU-positive, suggesting that a subset of retinal progenitors are expressing *prdm13* mRNA while still proliferating. As a whole, *prdm13* retinal expression in both species is similar.

GABAergic and glycinergic amacrine cells are reduced in *prdm13* ^−/−^ mouse retinas [34]. Our data revealed that only glycinergic amacrine cells are affected upon *prdm13* knockdown. This apparent discrepancy could highlight true differences between Prdm13 function in mouse and *Xenopus*. Alternatively, it could result from different experimental approaches since the *prdm13* ^−/−^ mouse is a null and we used a knockdown approach. Glycinergic amacrine cells may also be more sensitive to a reduction in Prdm13 protein level than GABAergic one. A CRISPR/Cas9 approach to target *prdm13* in *Xenopus* could contribute to address this hypothesis.

Our results indicate that Prdm13 negatively self-regulates its expression and that this may be due to a negative retro-control of Prdm13 on *ptf1a* expression. How Prdm13 negatively regulates *ptf1a* expression remains to be determined. Ptf1a levels have been shown to be regulated by autoregulation through the binding of Ptf1a, along with an E protein and Rbpj (the PTF1-J complex), to a conserved 2.3 kb sequence located 13.4 kb 5′ to the *ptf1a* coding region. This element has an enhancer activity in all *ptf1a* expression domains of the developing nervous system [53]. Therefore, one possibility is that Prdm13 negatively regulates *ptf1a* by blocking its autoregulation (Fig. 7c). This could be achieved through the enhancer element mentioned above for instance by preventing binding of the PTF1-J complex. Since Prdm13 has been shown to form complexes by protein-protein interactions with bHLH factors such as Ascl1 [22], an alternative model would be that Prdm13 binds to *Ptf1a* and converts the PTF1-J complex from an activator to a repressor. Further experiments are required to decipher Prdm13 mode of action in *Ptf1a* regulation. Other transcription factors, such as Satb2, Ebf3 and Neurod6, have been described as key regulators of amacrine subtype diversity [9]. The precise integration of Prdm13 function to this genetic network remains to be investigated.

Using whole-genome sequencing, it has recently been discovered that mutations in human *PRDM13* gene are associated with NCMD, a Mendelian form of human macular disease [35]. NCMD was initially considered as a slowly progressive disease, with many phenotypic similarities to age-related macular degeneration, including an abnormal accumulation of drusen, atrophy of the retinal pigment epithelium and overlying photoreceptor cells, choroidal neovascularization and loss of central vision. However, 20 years later it was realized that it is actually a nonprogressive developmental disorder, with highly variable expressivity [57]. A complete duplication of the *prdm13* gene was discovered in one family with NCMD. From gain of function analysis in the mouse [34] and our data in *Xenopus*, it is expected that increased level of Prdm13 in patients would likely lead to impaired amacrine cell fate specification. However, how this leads to macular degeneration remains unknown. A better knowledge of Prdm13 function in retinal development and maintenance should help unravelling the mechanisms by which *prdm13* mutations cause macular dystrophies.

## Conclusions

The present study confirms the important role of Prdm13 in amacrine cell subtype diversification downstream of Ptf1a. It also provides first evidence indicating that Prdm13 negatively regulates its expression, at least in part, by repressing Ptf1a in a feedback loop. Future studies, including the identification of its direct targets and partners, are required to determine how mechanistically Prdm13 control Ptf1a levels and promotes the generation of amacrine cell subtypes.

## Abbreviations

bHLH: Basic helix loop helix
CMZ: Ciliary marginal zone
CRISPR: Clustered Regularly Interspaced Short Palindromic Repeats
DOTAP: N-[1-(2,3-Dioleoyloxy)propyl]-N,N,N-trimethylammonium methyl-sulfate
EdU: 5-éthynyl-2′-déoxyuridine
GABA: Gamma-Aminobutyric acid
gad1: Glutamate decarboxylase 1
GCL: Ganglion Cell Layer38
glyt1: Glycine transporter 1
INL: Inner Nuclear Layer
MO: Morpholino Oligonucleotide
NCMD: North Carolina macular dystrophy
ONL: Outer Nuclear Layer
PR: PRDI-BF1-RIZ1
PRDM: PRDI-BF1 and RIZ homology domain
Ptf1a: Pancreas Specific Transcription Factor, 1a
RT-qPCR: Reverse transcriptase-quantitative polymerase chain reaction
SET: Su(var)3–9, Enhancer-of-zeste and Trithorax

## References

[1] MacNeil MA, Masland RH (1998). Extreme diversity among amacrine cells: implications for function. Neuron.

[2] MacNeil MA, Heussy JK, Dacheux RF, Raviola E, Masland RH (1999). The shapes and numbers of amacrine cells: matching of photofilled with Golgi-stained cells in the rabbit retina and comparison with other mammalian species. J Comp Neurol.

[3] Cherry TJ, Trimarchi JM, Stadler MB, Cepko CL (2009). Development and diversification of retinal amacrine interneurons at single cell resolution. Proc Natl Acad Sci U S A.

[4] Bassett EA, Wallace VA (2012). Cell fate determination in the vertebrate retina. Trends Neurosci.

[5] Goetz JJ, Farris C, Chowdhury R, Trimarchi JM (2014). Making of a retinal cell: insights into retinal cell-fate determination. Int Rev Cell Mol Biol.

[6] Cepko C (2014). Intrinsically different retinal progenitor cells produce specific types of progeny. Nat Rev Neurosci.

[7] Boije H, MacDonald RB, Harris WA (2014). Reconciling competence and transcriptional hierarchies with stochasticity in retinal lineages. Curr Opin Neurobiol.

[8] Voinescu PE, Emanuela P, Kay JN, Sanes JR (2009). Birthdays of retinal amacrine cell subtypes are systematically related to their molecular identity and soma position. J Comp Neurol.

[9] Kay JN, Voinescu PE, Chu MW, Sanes JR (2011). Neurod6 expression defines new retinal amacrine cell subtypes and regulates their fate. Nat Neurosci Nature Publishing Group.

[10] Feng L, Xie X, Joshi PS, Yang Z, Shibasaki K, Chow RL (2006). Requirement for Bhlhb5 in the specification of amacrine and cone bipolar subtypes in mouse retina. Development.

[11] Huang L, Hu F, Feng L, Luo X-J, Liang G, Zeng X-Y (2014). Bhlhb5 is required for the subtype development of retinal amacrine and bipolar cells in mice. Dev Dyn.

[12] Jiang H, Xiang M (2009). Subtype specification of GABAergic amacrine cells by the orphan nuclear receptor Nr4a2/Nurr1. J Neurosci.

[13] Elshatory Y, Everhart D, Deng M, Xie X, Barlow RB, Gan L (2007). Islet-1 controls the differentiation of retinal bipolar and cholinergic amacrine cells. J Neurosci.

[14] Jin K, Jiang H, Mo Z, Xiang M (2010). Early B-cell factors are required for specifying multiple retinal cell types and subtypes from postmitotic precursors. J Neurosci Society Neurosci.

[15] Ding Q, Chen H, Xie X, Libby RT, Tian N, Gan L (2009). BARHL2 differentially regulates the development of retinal amacrine and ganglion neurons. J Neurosci.

[16] Mo Z, Li S, Yang X, Xiang M (2004). Role of the Barhl2 homeobox gene in the specification of glycinergic amacrine cells. Development.

[17] Fog CK, Galli GG, Lund AH (2012). PRDM proteins: important players in differentiation and disease. BioEssays.

[18] Hohenauer T, Moore AW (2012). The Prdm family: expanding roles in stem cells and development. Development.

[19] Brzezinski JA, Lamba DA, Reh TA (2010). Blimp1 controls photoreceptor versus bipolar cell fate choice during retinal development. Development.

[20] Katoh K, Omori Y, Onishi A, Sato S, Kondo M, Furukawa T (2010). Blimp1 suppresses Chx10 expression in differentiating retinal photoreceptor precursors to ensure proper photoreceptor development. J Neurosci.

[21] Jung CC, Atan D, Ng D, Ploder L, Ross SE, Klein M (2015). Transcription factor PRDM8 is required for rod bipolar and type 2 OFF-cone bipolar cell survival and amacrine subtype identity. Proc Natl Acad Sci U S A.

[22] Chang JC, Meredith DM, Mayer PR, Borromeo MD, Lai HC, Ou Y-H (2013). Prdm13 mediates the balance of inhibitory and excitatory neurons in somatosensory circuits. Dev Cell.

[23] Hanotel J, Bessodes N, Thélie A, Hedderich M, Parain K, Van Driessche B (2014). The Prdm13 histone methyltransferase encoding gene is a Ptf1a-Rbpj downstream target that suppresses glutamatergic and promotes GABAergic neuronal fate in the dorsal neural tube. Dev Biol.

[24] Glasgow SM, Henke RM, Macdonald RJ, Wright CVE, Johnson JE (2005). Ptf1a determines GABAergic over glutamatergic neuronal cell fate in the spinal cord dorsal horn. Development.

[25] Huang M, Huang T, Xiang Y, Xie Z, Chen Y, Yan R (2008). Ptf1a, Lbx1 and Pax2 coordinate glycinergic and peptidergic transmitter phenotypes in dorsal spinal inhibitory neurons. Dev Biol.

[26] Hoshino M, Nakamura S, Mori K, Kawauchi T, Terao M, Nishimura YV (2005). Ptf1a, a bHLH transcriptional gene, defines GABAergic neuronal fates in cerebellum. Neuron.

[27] Pascual M, Abasolo I, Mingorance-Le Meur A, Martínez A, Del Rio JA, Wright CVE (2007). Cerebellar GABAergic progenitors adopt an external granule cell-like phenotype in the absence of Ptf1a transcription factor expression. Proc Natl Acad Sci U S A.

[28] Fujitani Y, Fujitani S, Luo H, Qiu F, Burlison J, Long Q (2006). Ptf1a determines horizontal and amacrine cell fates during mouse retinal development. Development.

[29] Nakhai H, Sel S, Favor J, Mendoza-Torres L, Paulsen F, Duncker GIW (2007). Ptf1a is essential for the differentiation of GABAergic and glycinergic amacrine cells and horizontal cells in the mouse retina. Development.

[30] Dullin J-P, Locker M, Robach M, Henningfeld K (2007). A, Parain K, Afelik S, et al. Ptf1a triggers GABAergic neuronal cell fates in the retina. BMC Dev Biol.

[31] Lelièvre EC, Lek M, Boije H, Houille-Vernes L, Brajeul V, Slembrouck A (2011). Ptf1a/Rbpj complex inhibits ganglion cell fate and drives the specification of all horizontal cell subtypes in the chick retina. Dev Biol Elsevier Inc.

[32] Jusuf PR, Harris WA (2009). Ptf1a is expressed transiently in all types of amacrine cells in the embryonic zebrafish retina. Neural Dev.

[33] Jusuf PR, Almeida AD, Randlett O, Joubin K, Poggi L, Harris WA (2011). Origin and determination of inhibitory cell lineages in the vertebrate retina. J Neurosci.

[34] Watanabe S, Sanuki R, Sugita Y, Imai W, Yamazaki R, Kozuka T (2015). Prdm13 regulates subtype specification of retinal amacrine interneurons and modulates visual sensitivity. J Neurosci.

[35] Small KW, DeLuca AP, Whitmore SS, Rosenberg T, Silva-Garcia R, Udar N (2016). North Carolina macular dystrophy is caused by Dysregulation of the retinal transcription factor PRDM13. Ophthalmology.

[36] Weleber RG (2016). Dysregulation of retinal transcription factor PRDM13 and North Carolina macular dystrophy. Ophthalmology.

[37] Yang Z, Tong Z, Chorich LJ, Pearson E, Yang X, Moore A (2008). Clinical characterization and genetic mapping of North Carolina macular dystrophy. Vis Res.

[38] Afelik S, Chen Y, Pieler T (2006). Combined ectopic expression of Pdx1 and Ptf1a/p48 results in the stable conversion of posterior endoderm into endocrine and exocrine pancreatic tissue. Genes Dev.

[39] Turner DL, Weintraub H (1994). Expression of achaete-scute homolog 3 in Xenopus embryos converts ectodermal cells to a neural fate. Genes Dev.

[40] Ohnuma S, Mann F, Boy S, Perron M, Harris WA (2002). Lipofection strategy for the study of Xenopus retinal development. Methods.

[41] Holt CE, Garlick N, Cornel E (1990). Lipofection of cDNAs in the embryonic vertebrate central nervous system. Neuron.

[42] Li M, Sipe CW, Hoke K, August LL, Wright MA, Saha MS (2006). The role of early lineage in GABAergic and glutamatergic cell fate determination in Xenopus Laevis. J Comp Neurol.

[43] Gleason KK, Dondeti VR, Hsia H-LJ, Cochran ER, Gumulak-Smith J, Saha MS (2003). The vesicular glutamate transporter 1 (xVGlut1) is expressed in discrete regions of the developing Xenopus Laevis nervous system. Gene Expr Patterns.

[44] Wester MR, Teasley DC, Byers SL, Saha MS (2008). Expression patterns of glycine transporters (xGlyT1, xGlyT2, and xVIAAT) in Xenopus Laevis during early development. Gene Expr Patterns.

[45] Parain K, Mazurier N, Bronchain O, Borday C, Cabochette P, Chesneau A (2012). A large scale screen for neural stem cell markers in Xenopus Retina. Dev Neurobiol.

[46] Lea R, Bonev B, Dubaissi E, Vize PD, Papalopulu N (2012). Multicolor fluorescent in situ mRNA hybridization (FISH) on whole mounts and sections. Methods Mol Biol.

[47] Inoue D, Wittbrodt J (2011). One for all-a highly efficient and versatile method for fluorescent immunostaining in fish embryos. PLoS One.

[48] Fischer AJ, Bosse JL, El-Hodiri HM (2013). The ciliary marginal zone (CMZ) in development and regeneration of the vertebrate eye. Exp Eye Res Elsevier Ltd.

[49] Perron M, Kanekar S, Vetter ML, Harris WA (1998). The genetic sequence of retinal development in the ciliary margin of the Xenopus eye. Dev Biol.

[50] Sandell JH, Martin SC, Heinrich G (1994). The development of GABA immunoreactivity in the retina of the zebrafish (brachydanio rerio). J Comp Neurol.

[51] Fry KR, Chen NX, Glazebrook PA, Lam DM (1991). Postnatal development of ganglion cells in the rabbit retina: characterizations with AB5 and GABA antibodies. Brain Res Dev Brain Res.

[52] Gábriel R (2000). Calretinin is present in serotonin- and gamma-aminobutyric acid-positive amacrine cell populations in the retina of Xenopus Laevis. Neurosci Lett.

[53] Meredith DM, Masui T, Swift GH, MacDonald RJ, Johnson JE (2009). Multiple transcriptional mechanisms control Ptf1a levels during neural development including autoregulation by the PTF1-J complex. J Neurosci.

[54] Nieber F, Pieler T, Henningfeld KA (2009). Comparative expression analysis of the neurogenins in Xenopus Tropicalis and Xenopus Laevis. Dev Dyn.

[55] Haley TL, Pochet R, Baizer L, Burton MD, Crabb JW, Parmentier M, et al. Calbindin D-28K immunoreactivity of human cone cells varies with retinal position. Vis Neurosci. 1995:12:301–7. 10.1017/s0952523800007987 7786851

[56] Haverkamp S, Müller U, Harvey K, Harvey RJ, Betz H, Wässle H (2003). Diversity of glycine receptors in the mouse retina: localization of the α3 subunit. J Comp Neurol.

[57] Small KW (1989). North Carolina macular dystrophy, revisited. Ophthalmol.

